# The Nutritional Environment Influences the Impact of Microbes on Drosophila melanogaster Life Span

**DOI:** 10.1128/mBio.00885-19

**Published:** 2019-07-09

**Authors:** Erin S. Keebaugh, Ryuichi Yamada, William W. Ja

**Affiliations:** a Department of Neuroscience, The Scripps Research Institute, Jupiter, Florida, USA; b Center on Aging, The Scripps Research Institute, Jupiter, Florida, USA; University of Texas Health Science Center at Houston

**Keywords:** *Drosophila*, life span, bacteria, microbiota, nutrition, nutritional environment, yeast

## Abstract

Microbes can extend Drosophila melanogaster life span by contributing to the nutritional value of malnourishing fly culture medium. The beneficial effect of microbes during malnutrition is dependent on their individual ability to proliferate in the fly environment and is mimicked by lifelong supplementation of equivalent levels of heat-killed microbes or dietary protein, suggesting that microbes can serve directly as a protein-rich food source. Here, we use nutritionally rich fly culture medium to demonstrate how changes in dietary composition influence monocolonized fly life span; microbes that extend fly life span on malnourishing diets can shorten life on rich diets. The mechanisms employed by microbes to affect host health likely differ on low- or high-nutrient diets. Our results demonstrate how *Drosophila*-associated microbes can positively or negatively influence fly life span depending on the nutritional environment. Although controlled laboratory environments allow focused investigations on the interaction between fly microbiota and nutrition, the relevance of these studies is not straightforward, because it is difficult to mimic the nutritional ecology of natural *Drosophila*-microbe interactions. As such, caution is needed in designing and interpreting fly-microbe experiments and before categorizing microbes into specific symbiotic roles based on results obtained from experiments testing limited conditions.

## Abstract (precis)

D. melanogaster ingests microorganisms growing within its rotting vegetation diet. Some of these microbes form associations with flies, while others pass through the gut with meals. Fly-microbe-diet interactions are dynamic, and changes to the fly culture medium can influence microbial growth in the overall environment. In turn, these alterations in microbial growth may not only impact the nutritional value of fly meals but also modulate behavior and health, at least in part due to direct contributions to fly nutrition. The interactive ecology between flies, microbes, and their environment can cause a specific microbe to be either beneficial or detrimental to fly life span, indicating that the environment should be considered a key influential factor in host-microbe interactions.

## OBSERVATION

Studies of Drosophila melanogaster have detailed pathways regulating the growth-promoting effects of microbes during development (1, 2) and identified key molecular and cellular changes associated with declining host-microbe homeostasis in aging hosts (3). Similar processes have since been described in juvenile and aged mammalian models (4, 5). While the fly-microbe model has made valuable scientific contributions, there is concern that contrasting results among recent studies will stall the progress and synthesis of future research (6).

The overall impact of microbes on *Drosophila* health remains unclear. It has been reported that association with microbes, or even species- or strain-specific associations, can extend or shorten fly life (6–13). The variability across studies may stem from the associated bacterial species or strains or from interactions between microbes and the different experimental conditions employed across studies.

Here, we resolve an interaction between two environmental factors, diet and microbes, to determine fly life span on high-nutrition diet. In consideration with our previous results (10, 12), we demonstrate a nutrition-dependent shift in the role of specific microbes—from beneficial to deleterious—on *Drosophila* longevity. It is therefore crucial that future fly-microbe investigations account for the dynamic and interrelated nature of flies, microbes, and their diet. How dietary conditions might influence microbe-associated phenomena should be carefully considered when designing experiments and interpreting results; although we focus here on fly life span, environmental and microbial interconnections may have far-reaching effects on fly behavior or physiology and potentially impact fly fitness in nature. Ecologically relevant, integrative studies on the fly microbiome may be needed to allow for a better understanding of how extrinsic factors influence the outcome of *Drosophila*-microbe interactions.

## 

### Deleterious effect of microbes on high-nutrition diet.

**FIG 1:**
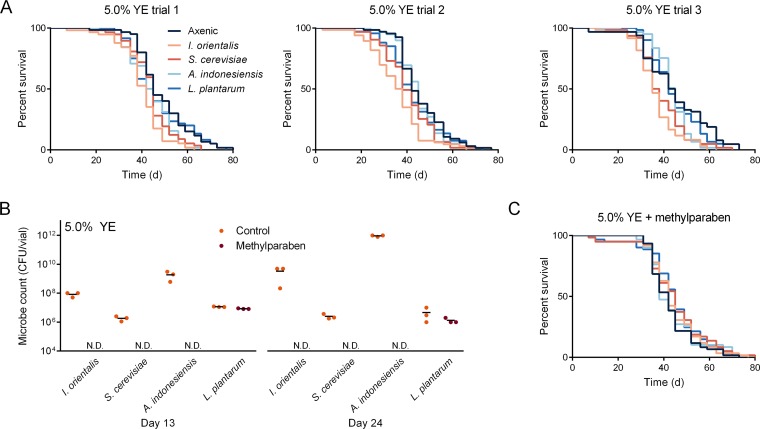
*I. orientalis* is deleterious to fly longevity in a high-nutrition environment (5.0% YE). (A) Three independent survival trials of axenic or monoxenic flies inoculated with one dose of live microbes (trial 1, 5 × 10^5^ CFU/vial *I. orientalis* and S. cerevisiae, 5 × 10^7^ CFU/vial *A. indonesiensis* and *L. plantarum*; trials 2 and 3, 1.5 × 10^5^ CFU/vial *I. orientalis* and S. cerevisiae, 1.5 × 10^7^ CFU/vial *A. indonesiensis* and *L. plantarum*). In trials 1 and 2, only *I. orientalis* association resulted in a change in median life span compared to the axenic control (trial 1, *P = *0.0055; trial 2, *P = *0.0017; *P* values by Fisher’s exact test). In trial 3, both *I. orientalis* and S. cerevisiae reduced median life span compared to the axenic control (*I. orientalis*, *P = *1.0 × 10^−4^; S. cerevisiae, *P = *0.049, *P* values by Fisher’s exact test). Association with *I. orientalis* and S. cerevisiae shortened life span compared to the axenic control (*I. orientalis* versus axenic control, trial 1, *P = *1.0 × 10^−4^; trial 2, *P = *2.9 × 10^−5^; trial 3, *P = *1.0 × 10^−4^, and S. cerevisiae versus axenic control, *P = *0.015; *P* values by log rank test). Other microbes failed to show a consistent effect. Time is shown in days (d) on the *x* axes. (B) Methylparaben eliminates microbes from the fly environment. Flies were maintained on diets with 0.3% methylparaben or without methylparaben (control diets), and microbes were collected from fly enclosures on days 13 and 24 (3 or 4 days after the transfer to fresh food). Average microbe counts are indicated by horizontal bars calculated from three vials (N.D., not detected). Axenic spent vials produced no microbe counts at any time (data not shown). (C) Methylparaben supplementation rescues the shortened life span induced by *I. orientalis*, resulting in identical survival compared to the axenic control (*P = *1.0 by log rank test). A total of 57 to 67 flies were used for life span studies in each treatment. The experiments shown in trial 1 of panel A and panels B and C were performed at the same time.

Although microbes were beneficial to fly life span on nutrient-poor medium (0.1% yeast extract [YE]) (10, 12) and had no effect on the life span-maximized diet (0.5% YE) (12), Issatchenkia orientalis association shortened longevity on high-nutrition diet (5.0% YE) compared to the axenic control (Fig. 1A). Association with Saccharomyces cerevisiae sometimes resulted in statistically different survival and median life span, but only I. orientalis showed a consistent effect across multiple trials. Methylparaben supplementation eliminated the presence of most microbes (14) (Fig. 1B) and suppressed the negative impact of *I. orientalis* on longevity in a high-nutrition environment (Fig. 1C), suggesting that a continuous microbial presence is required for shortening life.

**FIG 2:**
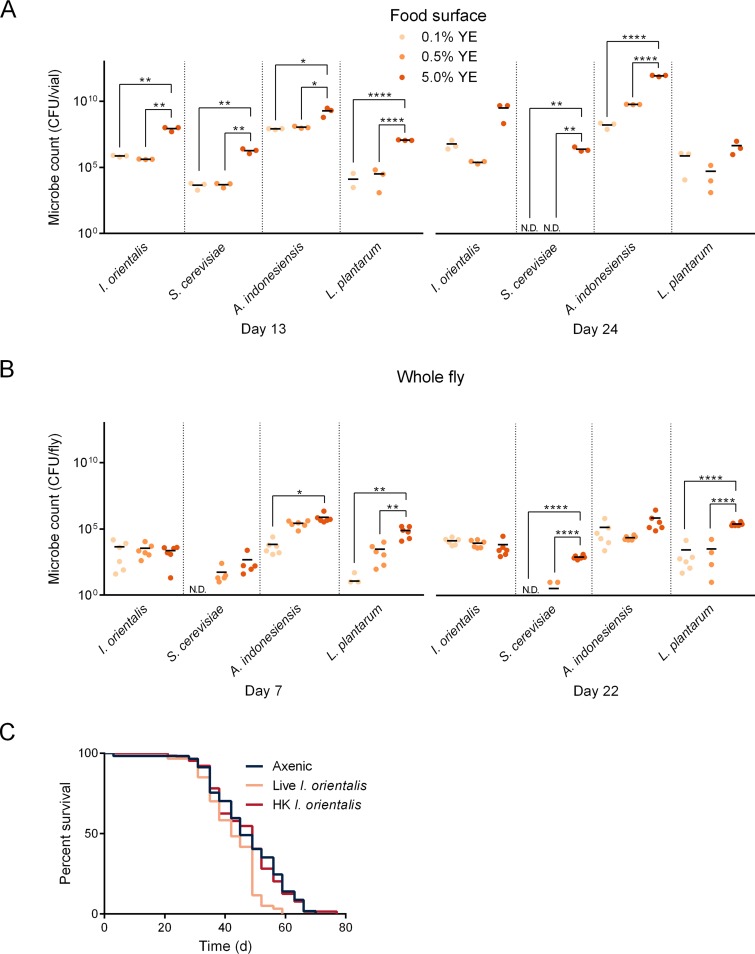
Microbes thrive on high-nutrition diets, and live *I. orientalis* is required for detrimental effects on host life span. (A) Environmental microbe counts increase on higher-nutrient diets. Microbes were collected on days 13 and 24 (3 or 4 days after the transfer to fresh food), and average microbe counts from the fly enclosure are indicated by horizontal bars calculated from three vials per condition. (B) Increased environmental microbe numbers do not necessarily result in higher fly internal microbial load. While S. cerevisiae, A. indonesiensis, and *L. plantarum* show highest microbial load on the high-nutrient diet, *I. orientalis* maintains consistent numbers. Flies were harvested on days 7 and 22 (3 or 4 days after the transfer to fresh food), and average microbe counts from whole flies are indicated by horizontal bars calculated from six individual flies per condition. Missing values are equal to zero and are included in the average calculation. Values that are significantly different by one-way ANOVA followed by Tukey’s multiple-comparison test are indicated by bars and asterisks as follows: *, *P < *0.05; **, *P < *0.01; ****, *P < *0.0001. N.D., not detected. Axenic control flies and their spent vials produced no microbe counts at any time (data not shown). (C) The detrimental effect of *I. orientalis* on fly life span is abolished when providing heat-killed (HK) microbes (axenic control versus live *I. orientalis*, *P = *0.0021; axenic control versus HK *I. orientalis*, *P = *1.0; *P* values by log rank test). Flies on 5.0% YE diet were either inoculated with live *I. orientalis* once as young adults (5 × 10^5^ CFU/vial) or provided with HK microbes throughout life (3 × 10^8^ CFU/vial every 3 or 4 days, which is similar to the average daily level of day 13 measures in panel A). A total of 57 to 64 flies were studied in each treatment. The experiments shown in panels A and B were performed at the same time as trial 1 in Fig. 1A and the experiments shown in Fig. 1B and C.

All microbes showed greater numbers in the environment on the high-nutrition diet (Fig. 2A), although this did not always lead to increased fly internal microbial load (Fig. 2B). To determine whether microbes exacerbate the detrimental effect of high-nutrient diet by further adding to the nutritional value of the 5.0% YE food, we examined the effect of heat-killed microbes dosed similarly to the average amount of *I. orientalis* measured on 5.0% YE food surfaces at day 13 (Fig. 2A). Lifelong supplementation with heat-killed *I. orientalis* has no effect on fly life span on high-nutrition diet compared to flies inoculated once with live microbes (Fig. 2C). Therefore, whereas an active microbial metabolism is not essential for fly life span extension on malnutrition diet (10, 12), reduced longevity on high-nutrition food requires live microbes. These results suggest that microbes affect fly life span in different nutritional environments using distinct mechanisms.

### Discussion.

In the diet used in our study, increasing nutrition (yeast extract) often resulted in a greater number of microbes in the fly environment, but not in whole flies, potentially because microbes predominantly reside on the food while only a few microbes also stably colonize the gut (15, 16). For S. cerevisiae, Acetobacter indonesiensis, and Lactobacillus plantarum, increased numbers in the environment are generally associated with greater fly internal load. Their lack of an effect on life span for flies on a high-nutrition diet indicates that flies have an increased tolerance to these three microbes, as measured by fly survival. Conversely, the unchanged numbers of internal *I. orientalis* across diets—in the face of greater environmental numbers on high-nutrition food that likely lead to greater ingestion of microbes—suggest an ability to clear this specific microbe. Microbe clearance could result from a variety of factors, including increased host resistance, digestion, or microbe excretion rates through the gut. A heightened response to *I. orientalis* may explain this microbe’s specific deleterious effect on fly longevity; previous studies have linked chronic activation of immune pathways with reduced life span (17). The requirement for live *I. orientalis* suggests that the effect on high-nutrition diet relies on an active microbial metabolism or on heat-labile molecules; both possibilities are distinct from the effects on malnourished fly longevity, where heat-killed microbes are sufficient (10, 12). Importantly, our results show that a single microbial species can be beneficial or deleterious to fly life span, depending on the nutritional environment, highlighting the importance of considering the diet before categorizing the symbiotic relationship between a host and a specific microbe (e.g., pathogenic or commensal).

Although we previously identified protein nutrition as the mechanism for microbial influences on malnutrition diet, other nutrients or factors will likely contribute in other conditions (10, 18–21). The ultimate impact of microbes on fly life span will likely be the sum of their effects on nutrition and other host processes such as immunity, and these parameters will be influenced by interacting environmental factors. Previous studies of *Drosophila* and microbes have shown variable effects on fly life span. In light of our results, the different diets employed, the particular microbes present, and the propensity for those microbes to proliferate in the fly environment may explain the contrasting findings. We suspect that many studies implement conditions where the effects of microbes on longevity are obscured by the diets and microbes used in each laboratory. The influence of microbes on nutrition and the interaction of microbes with particular nutritional environments must be considered in studies where the microbiota of *Drosophila*, and other animals (7, 22–24), is claimed to play a role. Careful consideration of the fly ecosystem will optimize the use of the fly-microbe model in future investigations of host-microbe biology.

### Data and materials. (i) Fly strains.

Axenic Drosophila melanogaster Dahomey flies were generated by bleach treatment of embryos as previously described (12). Experiments in this report used first-generation axenic subjects.

**(ii) Diets.** Standard stock food contained 5.0% sucrose, 1.5% dry active yeast, 5.0% cornmeal, 1.5% agar (wt/vol), 0.4% propanoic acid, and 0.035% phosphoric acid (vol/vol) and was used to feed the flies until they were placed on experimental diets. Experimental diets contained the same acid preservatives and sucrose amounts with 5.0% yeast extract (YE), 8.6% cornmeal, and 0.5% agar (wt/vol). Variations in YE concentration were used as indicated in Fig. 2. Methylparaben (methyl 4-hydroxybenzoate) diets contained 0.3% (wt/vol) methylparaben diluted from a 10% (wt/vol) stock in 100% ethanol (EtOH) instead of acid preservatives. All experimental diets were autoclaved at 121°C for 30 min, and acids or methylparaben were added after cooling to below 65°C. For life span studies, experimental diets were dispensed into pre-autoclaved vials at a volume of 2 ml/vial.

**(iii) Microbial strains.** *Issatchenkia orientalis*, Saccharomyces cerevisiae, Acetobacter indonesiensis SB003, and Lactobacillus plantarum SB001 were grown in MRS broth and described previously (12). Microbes were heat-killed by a 30-min 121°C autoclave cycle, aliquoted, and stored at −20°C for subsequent use.

**(iv) Life span.** Male flies aged 3 to 5 days old were transferred to vials containing experimental diets at approximately 20 flies/vial and maintained in incubators set at 25°C, a 12-h/12-h light/dark cycle, and 60% humidity. Flies were transferred to fresh food every 3 or 4 days. Trial 1 in Fig. 1A used flies inoculated as adults, and trials 2 and 3 used flies inoculated as embryos.

**(v) Microbial load measurements.** To measure the microbial load of adults, flies were sterilized with 70% ethanol, washed three times with sterile 1× PBS, and single flies were homogenized in 100 μl of 1× PBS for 10 s. To measure environmental microbe growth, spent food vials were rinsed with 1 ml of 1× PBS. Microbial counts were quantified by serial dilution plating of either homogenized fly samples or vial washes on MRS agar. A portion of the *I. orientalis* data in Fig. 2B was from Yamada et al. (12).

**(vi) Statistics.** Differences in microbe counts were determined by one-way analysis of variance (ANOVA). Each strain and day was analyzed separately, and multiple comparisons were accounted for using Tukey’s multiple-comparison test. Differences between survival curves using the log rank test and median life spans using Fisher’s exact test were analyzed using OASIS 2 (25). Bonferroni *P* values were reported for log rank tests.
